# Effectiveness and cost-utility of a guided self-help exercise program for patients treated with total laryngectomy: protocol of a multi-center randomized controlled trial

**DOI:** 10.1186/s12885-016-2613-6

**Published:** 2016-08-02

**Authors:** Femke Jansen, Ingrid C. Cnossen, Simone E. J. Eerenstein, Veerle M. H. Coupé, Birgit I. Witte, Cornelia F. van Uden-Kraan, Patricia Doornaert, Weibel W. Braunius, Remco De Bree, José A. U. Hardillo, Jimmie Honings, György B. Halmos, C. René Leemans, Irma M. Verdonck-de Leeuw

**Affiliations:** 1 Department of Otolaryngology-Head and Neck Surgery, Cancer Center Amsterdam (CCA), VU University Medical Center, PO Box 7057, 1007 MB Amsterdam, The Netherlands; 2 Department of Epidemiology and Biostatistics, VU University Medical Center, PO Box 7057, 1007 MB Amsterdam, The Netherlands; 3 Department of Clinical Psychology, Vrije Universiteit Amsterdam, Van der Boechorststraat 1, 1081 BT Amsterdam, The Netherlands; 4 Department of Radiation Oncology, VU University Medical Center, PO Box 7057, 1007 MB Amsterdam, The Netherlands; 5 Department of Head and Neck Surgical Oncology, UMC Utrecht Cancer Center, Heidelberglaan 100, 3584 CX Utrecht, The Netherlands; 6 Department of Otorhinolaryngology, Erasmus MC, University Medical Center, PO Box 2040, 3000 CA Rotterdam, The Netherlands; 7 Department of Otorhinolaryngology-Head and Neck Surgery, Radboud University Nijmegen Medical Center, PO Box 9101, 6500 HB Nijmegen, The Netherlands; 8 Department of Otorhinolaryngology, Head and Neck Surgery, University of Groningen, University Medical Center Groningen, PO Box 30.001, 9700 RB Groningen, The Netherlands

**Keywords:** Exercise program, Total laryngectomy, Head and neck cancer, Swallowing problems, Speech problems, Shoulder problems

## Abstract

**Background:**

Total laryngectomy with or without adjuvant (chemo)radiation often induces speech, swallowing and neck and shoulder problems. Speech, swallowing and shoulder exercises may prevent or diminish these problems. The aim of the present paper is to describe the study, which is designed to investigate the effectiveness and cost-utility of a guided self-help exercise program built into the application “In Tune without Cords” among patients treated with total laryngectomy.

**Methods/design:**

Patients, up to 5 years earlier treated with total laryngectomy with or without (chemo)radiation will be recruited for participation in this study. Patients willing to participate will be randomized to the intervention or control group (1:1). Patients in the intervention group will be provided access to a guided self-help exercise program and a self-care education program built into the application “In Tune without Cords”. Patients in the control group will only be provided access to the self-care education program. The primary outcome is the difference in swallowing quality (SWAL-QOL) between the intervention and control group. Secondary outcome measures address speech problems (SHI), shoulder disability (SDQ), quality of life (EORTC QLQ-C30, QLQ-H&N35 and EQ-5D), direct and indirect costs (adjusted iMCQ and iPCQ measures) and self-management (PAM). Patients will be asked to complete these outcome measures at baseline, immediately after the intervention or control period (i.e. at 3 months follow-up) and at 6 months follow-up.

**Discussion:**

This randomized controlled trial will provide knowledge on the effectiveness of a guided self-help exercise program for patients treated with total laryngectomy. In addition, information on the value for money of such an exercise program will be provided. If this guided self-help program is (cost)effective for patients treated with total laryngectomy, the next step will be to implement this exercise program in current clinical practice.

**Trial registration:**

NTR5255 Protocol version 4 date September 2015.

## Background

In the Netherlands, approximately 700 persons are diagnosed with laryngeal cancer each year and an additional 600 persons are diagnosed with hypopharyngeal cancer [1]. Depending on primary tumor location and tumor size, patients with laryngeal cancer are treated with (laser)surgery or (chemo)radiation preserving the larynx or, in case of advanced tumors or tumors with extra laryngeal growth, treated with total laryngectomy (TL) (i.e. resection of the entire larynx). Hypopharyngeal cancer with laryngeal involvement can also be treated with TL. About 150 patients are treated with TL each year either as primary treatment, often in combination with adjuvant (chemo)radiation, or for recurrent disease after prior larynx-preserving treatment (salvage laryngectomy) [2].

Patients treated with total laryngectomy often report speech, swallowing and shoulder problems [3–6]. These problems may give rise to emotional distress and decrease quality of life of patients [7, 8]. Scores on several quality of life scales, such as dyspnoea, speech and social contact, are still diminished 1 year after TL [9].

Several studies investigated the effectiveness of swallowing and shoulder exercises targeting swallowing and shoulder problems in head and neck cancer patients [10–27]. Most of these studies hypothesized that performing exercises may prevent or diminish swallowing [11–16, 21–26] and shoulder problems [17–19, 27]. However, studies targeting swallowing problems focused on patients treated with primary (chemo)radiation (except for two studies [10, 26]), while studies targeting shoulder problems focused on patients treated with a neck dissection with or without (chemo)radiation [17–19, 27]. No studies have primarily focused on patients treated with TL with or without (chemo)radiation.

Therefore, based on previous experience [28], a guided self-help program has been developed using a participatory design approach targeting patients previously treated with TL. Such a guided self-help program is expected to be easily accessible for patients and limits burden on time of healthcare providers [29]. In the first development phase [30], a needs assessment was performed by means of one focus group in patients and their partners (*n* = 9) and four multidisciplinary meetings with healthcare professionals involved in (after)care for patients treated with TL (*n* = 11). It was found that a self-help application should not only include an exercise program, but also information modules comprising of daily care for the tracheostomy or voice prosthesis, nutrition, smelling and speech quality.

In the second phase [30], a prototype of the newly developed self-help application, including an exercise program and self-care education program (i.e. information modules) was developed. The usability of the self-help application was evaluated by four patients treated with TL and ten speech therapists of different head and neck cancer centers in the Netherlands. Based on these findings, further improvements were made to the application, which was named “In Tune without Cords (ITwC)”.

Subsequently, the self-care education program of this final application was tested on its feasibility in a group of patients that underwent TL up to 2 years ago. It was found that the usage rate of the self-care education program was good and patients were satisfied with the application [31].

The present study aims to investigate the effectiveness and cost-utility of the guided self-help exercise program built into ITwC to prevent or diminish speech, swallowing and neck and shoulder problems in patients treated with TL. This paper describes the study design, expected results and benefit of the study.

## Methods

### Design

**Fig. 1:**
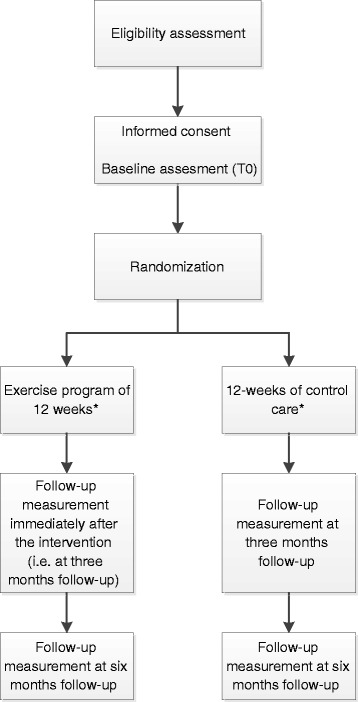
Design of the randomized controlled trial

This study is a prospective multi-center randomized controlled trial with two parallel groups, comparing an intervention group to a control group with equal (1:1) randomization. Patients will be recruited from various head and neck cancer centers in the Netherlands in which TLs are performed. Patients in all participating centers who are found eligible will be asked to participate in this study by their treating head and neck surgeon, speech therapist or nurse specialist/practitioner at the last consultation prior to discharge, at follow-up visit or by mail plus telephone. After signing informed consent, patients will be randomly assigned to the intervention or control group. The baseline assessment takes place before randomization, with follow-up assessment immediately after the intervention or control period (i.e. at 3 months follow-up) and at 6 months follow-up (Fig. 1). Reasons for drop-out will be registered. This study has been approved by the Medical Ethics Committee of the VU University Medical Center.

### Study sample

Patients up to 5 years earlier treated with TL with or without (chemo)radiation will be asked to participate in this study. The time period of up to 5 years is chosen, since it is hypothesized that patients recently treated with TL as well as patients treated longer ago may benefit from the intervention. Up to 5 years, all patients treated with TL regularly visit the hospital for follow-up visits. Patients will be excluded from participation when they are treated with total laryngectomy combined with total glossectomy, are treated with a partial laryngectomy, are younger than 18 years, have cognitive impairments, and/or are unable to understand the Dutch language.

### Randomization

Patients will be randomized to the intervention or control group by an independent person, stratified for 1) time since TL (≤6 months after TL vs. > 6 months after TL) to distinguish patients who are recently treated and who may in some cases receive postoperative (chemo)radiation from patients who completed their treatment longer ago; 2) neck dissection (not treated with a neck dissection versus treated with a neck dissection); and 3) TL indication (primary TL or salvage TL) in blocks of two and four using an automatically created randomization list. Although this is a multicenter study, we chose not to stratify for study center, because head and neck cancer care is mainly centered in the University Hospitals in the Netherlands and highly protocolled via the Dutch Head and Neck Oncology Cooperative Group (NWHHT).

Patients randomized to the intervention group will be provided access to the guided self-help exercise program targeting speech, swallowing and shoulder problems built into the application ITwC as well as the self-care education program. Patients randomized to the control group will, in addition to usual care, also be provided access to the self-care education program, since we aim to investigate the effectiveness of the guided self-help exercise program only.

The guided self-help program ITwC will be provided by Internet [32] or as booklet plus DVD. Patients can choose themselves between these two formats. Both formats contain the same content (i.e. the same written instructions, images and videos are available).

### Self-help exercise program

**Fig. 2:**
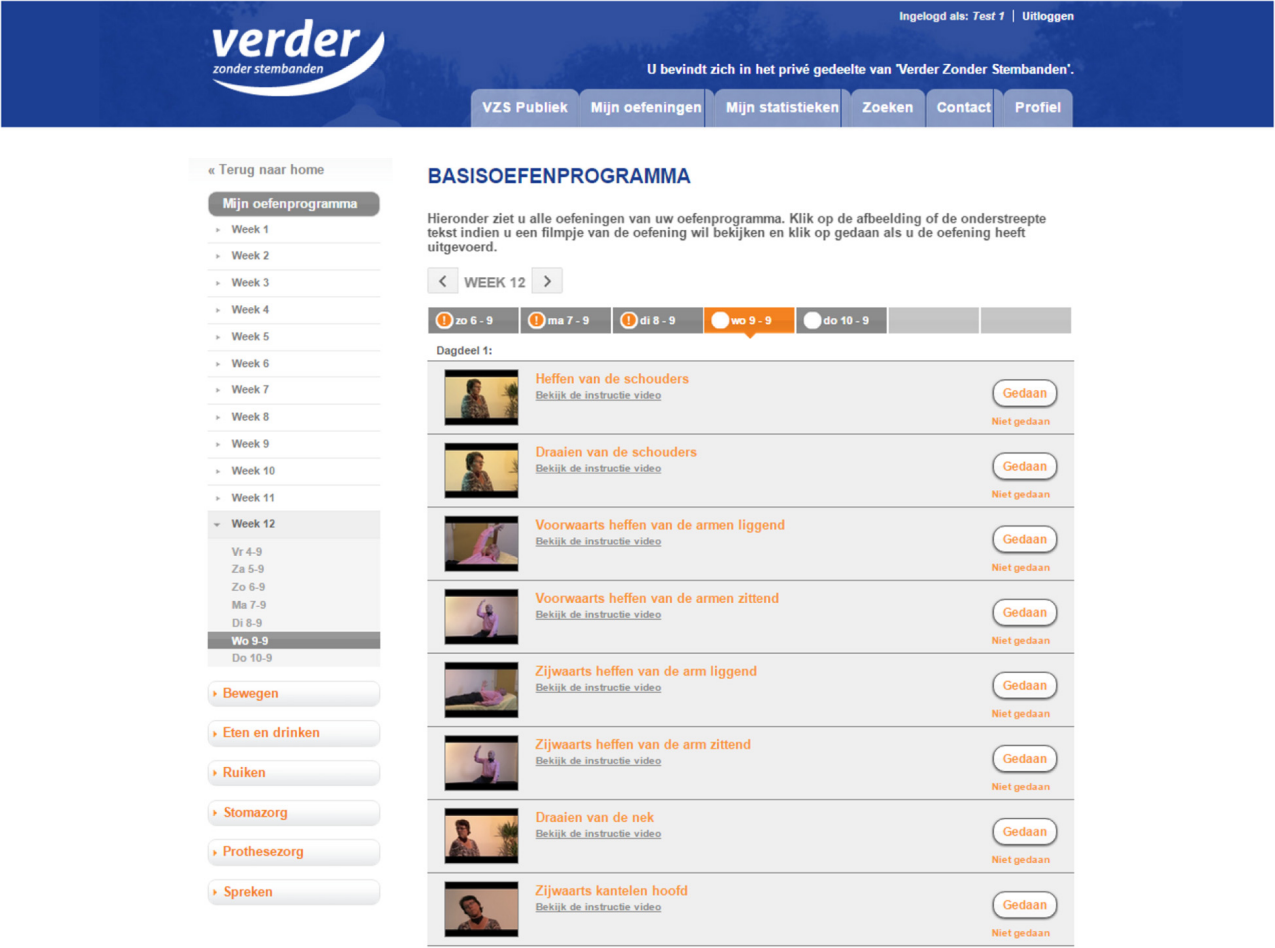
Screenshot self-help exercise program

The guided self-help exercise program consist of three flexibility exercises for head and neck (e.g., turning the head), four exercises for the shoulders (e.g., raising the shoulders) and eight range-of-motion exercises for the tongue, lips, and jaw targeting speech and swallowing problems (e.g., moving jaw with open mouth) aiming to prevent or diminish speech, swallowing and shoulder problems (Fig. 2). In addition, for patients with facial lymphedema (diagnosed by their head and neck surgeon), five additional exercises are provided (e.g., massaging the facial and jaw area).

Patients randomized to the intervention group will be invited by their speech therapist or nurse practitioner/specialist for a consultation of half an hour in the hospital during which the patient will be instructed on how to perform the prescribed exercises. Following baseline consultation, patients are asked to perform the exercises at home, three times a day for 12 weeks, using the written instructions, images and videos of the prescribed exercises built into the online application of ITwC or a booklet plus DVD. The prescribed exercises and intensity of the exercises are fixed. The entire exercise protocol takes approximately 10–15 min a time.

To enhance exercise adherence, patients are asked to fill out a diary during the 12 week period. In addition, patients will be coached on a weekly basis by their speech therapist or nurse practitioner/specialist via e-mail or telephone. During these coaching sessions, patients will be asked on their general well-being and exercise performance. The speech therapist or nurse practitioner/specialist will take notes of these sessions in order to assess barriers and facilitators of adherence to the exercise program.

### Self-care education program

**Fig. 3:**
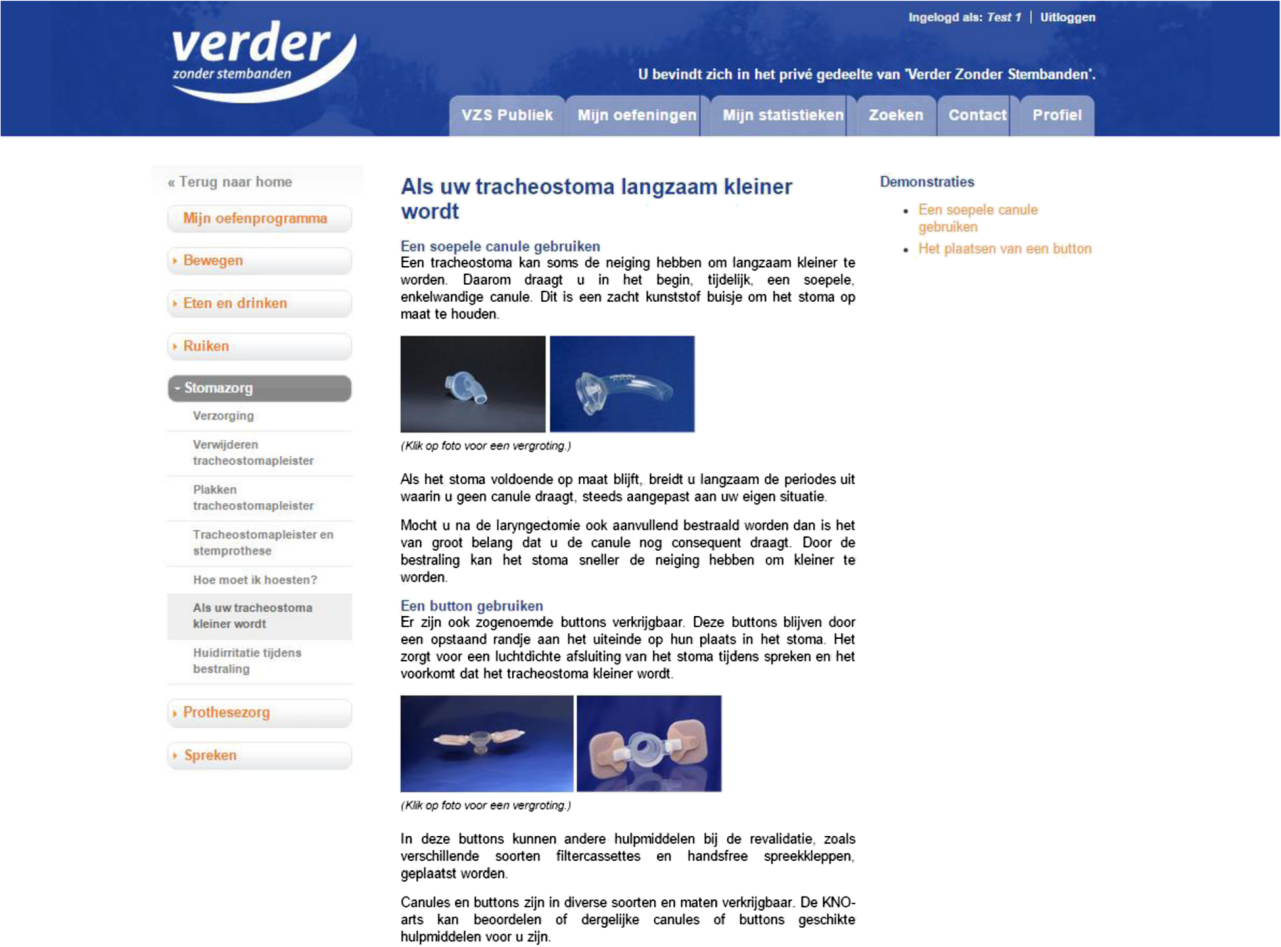
Screenshot self-care education program

The self-care education program of ITwC provides information and self-care advice on stoma care, voice prosthesis care, speech, smelling, nutrition and mobility (Fig. 3). The information and self-care advice are provided in written text and are supported by several images and videos. Patients randomized to the intervention and control group are both provided with the self-care education program; patients can decide whether they are interested in using it.

### Outcome assessment

For this study, different patient reported outcome measures will be collected at baseline, immediately after the intervention or control period (i.e. at 3 months follow-up) and at 6 months follow-up. Completion of the outcome measures is expected to take on average 30 min.

#### Primary outcome measure

The primary outcome measure of this study is the swallowing quality of life questionnaire (SWAL-QOL). The SWAL-QOL is a 47-item patient-reported outcome measure that consists of ten different quality of life domains namely Food selection (two items), Eating duration (two items), Eating desire (three items), Fear (four items), Burden (two items), Mental health (five items), Social functioning (five items), Communication (two items), Sleep (two items) and Fatigue (three items). Furthermore, a symptom scale (14 items) is included. Based on the 30-items of the first seven mentioned quality of life domains a total SWAL-QOL score can be calculated. All SWAL-QOL scales range from 0 to 100, a higher score indicates more impairment. Finally, three separate questions on nutrition intake (normal, soft, pureed, mostly tube feeding, only liquids, tube feeding solely), liquids intake (all liquids, thick liquids, very thick liquids, thickened liquids, no liquids), and general health (poor, moderate, good, very good, excellent) are included. The SWAL-QOL has been translated into Dutch and validated for patients with head and neck cancer [33] and laryngeal cancer [8].

#### Secondary outcome measures

Secondary outcome measures of this study are the speech handicap index (SHI), the shoulder disability questionnaire (SDQ), the European Organization for Research and Treatment of Cancer generic and head and neck cancer specific quality of life measures (EORTC QLQ-C30 and EORTC QLQ-H&N35) and the Patient Activation Measure (PAM). In addition, the EuroQol-5 dimensions (EQ-5D) and patient-reported outcome measures on healthcare usage and productivity losses will be assessed to enable a cost-utility analysis.

The SHI is a 30-item patient reported outcome measure on speech problems in daily life. Response categories range on a five point scale (never, almost never, sometimes, almost always, always). In addition, the questionnaire includes an overall speech quality item, with four response categories (good, reasonable, poor, severe). A total SHI score can be calculated by summing all items (score ranges from 0 to 120), with higher scores indicating higher levels of speech-related problems. The SHI had been translated into Dutch and validated for use among head and neck cancer patients [34] and laryngeal cancer patients [8].

The SDQ is a validated pain related disability outcome measure including 16 items describing common conditions that may induce symptoms in patients with disorders of the shoulder [35]. All items refer to the preceding 24 h. Options are “yes”, “no” and “not applicable”. The “not applicable” category should be used when the condition referred to has not occurred during the preceding 24 h. A final score will be calculated by dividing the number of “yes” scored items by the total number of items applicable and multiplying this score by 100. The final score ranges from 0 (no disability) to 100 (all applicable items scored “yes”), in which a higher score indicates greater impairment.

The EORTC QLQ-C30 is a cancer-specific quality of life measure developed for repeated assessments within clinical trials. It is developed in a cross-cultural setting and is a valid and reliable instrument for quality of life assessments in various cancer populations, including head and neck cancer patients [36]. It contains five functional scales (physical, cognitive, emotional, social, role), a global quality of life scale, three symptom scales (pain, fatigue and nausea/vomiting) and six single items (dyspnoea, insomnia, loss of appetite, constipation, diarrhea and financial difficulties). All scales and single items can be converted to a score from 0 to 100. A higher score on the functioning scales or the global quality of life scale represents a better quality of life, whereas a higher score on the symptoms scales or the single items indicate higher level of symptoms.

The EORTC QLQ-H&N35 is a cancer-specific module developed for quality of life assessments in head and neck cancer patients [37] in conjunction with the EORTC QLQ-C30. It contains seven symptom scales (pain, swallowing, senses, speech, social eating, social contact and sexuality), six symptom items (teeth, trismus, dry mouth, sticky saliva, cough and feeling ill) and five additional items concerning the use of painkillers, nutritional supplements and feeding tube and weight loss or gain. All scales and single items range in score from 0 to 100, higher scores indicate a higher level of symptoms.

The PAM is a 13-item patient reported outcome measure on self-reported knowledge, skills and confidence for self-management of one’s health or chronic condition [38]. Patients are asked to report their level of agreement with various statements on a four point scale (e.g. strongly disagree, disagree, agree, strongly agree) or to indicate that the item was not applicable. A total score can be calculated by calculating a mean score of all the applicable items (items which were answered on the four point scale), which is transformed to a standardized activation score ranging from 0 to 100.

The EQ-5D consists of five items measuring problems on five dimensions of quality of life (mobility, self-care, usual activities, pain/discomfort and anxiety/depression). Patients can answer they have no problems, some problems or extreme problems [39]. The resulting profile of answers (one of 243 possibilities) can be transformed to a utility given by the general public: the EQ-5D index using the Dutch index tariff [40]. Furthermore, a visual analogue scale is included, which represents the patient’s judgment of his own health state on a scale from 0 (worst health state) to 100 (best health state).

Direct medical (healthcare and medication use), direct non-medical (travelling costs and help received from family or friends) and indirect non-medical costs (productivity losses) in the previous 3 months will be measured using an adapted version of the medical consumption questionnaire (iMCQ) [41] and productivity cost questionnaire (iPCQ) [42] of the Institute for Medical Technology Assessment of the Erasmus University Rotterdam, the Netherlands, as recommended in the Dutch Health Care Insurance Board (CVZ) guideline [43].

#### Sociodemographic and medical data

Sociodemographic characteristics (age, gender, education level and living situation) will be assessed at baseline using a study-specific questionnaire. Clinical characteristics including information on cancer stage (TNM classification) and cancer treatment, time since TL and co-morbidity will be collected from the hospital information system using a study-specific case report form. Co-morbidity will be measured using the Adult Comorbidity Evaluation 27 (ACE-27), in which patients can be classified into one of four grades of comorbidity (none, mild, moderate or severe) [44]. Besides, a case report form on health care use in the hospital during the study period, including visits to medical specialists, day treatment (e.g. chemotherapy) and hospital admission, will be completed using the hospital information system.

### Sample size

In a previous study of Rinkel et al. [8] a standard deviation of 21 on the SWALQOL, the primary outcome measure in this study, was found. In addition, a difference in improvement of 12 points was found to be clinically meaningful. Based on a power of 80 %, a significance level of 5 % and a standard deviation of 21, in total 100 patients are needed (50 patients per intervention arm), to demonstrate a difference in improvement of 12 points between the intervention and control group at 6 months follow-up. Results will be analyzed according to the intention-to-treat principle and all drop-outs, when possible, will be approached for follow-up measurements.

### Statistical analyses

All analyses will be performed using the IBM Statistical package for the Social Science (SPSS) version 22 (IBM Corp., Armonk, NY USA) and STATA version 12.1 (StataCorp LP, Texas, USA). Descriptive statistics (e.g. frequencies, percentages, means and standard deviations or medians and (interquartile) ranges) will be generated for all socio-demographic and clinical characteristics and outcome measures. Chi-square tests, independent *t*-tests and Mann-Whitney tests (in case of non-normality of the measure) will be used to analyze whether randomization resulted in comparable patient groups. Analyzes will be performed according to the intention-to-treat principle. A *p*-value < .05 will be considered significant.

Independent *t*-tests will be used to measure differences in swallowing quality (SWAL-QOL), speech quality (SHI), shoulder function (SDQ) and quality of life (EORTC QLQ-C30 and H&N35) between the intervention and control group at follow-up measurements. Linear mixed models and generalized estimating equations, with fixed effects for group and measurement and their two-way interaction and a random effect for subject, will be used to compare longitudinal changes in both groups over time.

An incremental cost utility ratio (ICUR) will be calculated measuring the cost per gained quality-adjusted life year (QALY). The ICUR will be calculated by dividing the incremental costs by the incremental QALYs, using the formula: ICUR = (C_int_ - C_con_)/(QALY_int_ - QALY_con_). Total costs will be calculated using a societal perspective, including intervention costs, direct medical, direct non-medical and indirect non-medical costs. Direct medical and non-medical costs will be calculated by multiplying resource use by integral cost prices as presented in the CVZ guideline on cost studies [43]. Indirect non-medical costs will be calculated using the friction cost approach as recommended in the CVZ guideline [43]. Total QALYs will be calculated by using the utility scores linked to the various health states of the EQ-5D [40]; in essence the length of time a patient spends in a particular health condition is weighed by the corresponding utility. Missing data on direct medical, direct non-medical and indirect non-medical costs measured using the cost questionnaire and utilities measured using the EQ-5D will be imputed using multiple imputation. If necessary, the estimates of the cost difference, the QALY difference and the ICUR will be corrected for differences in baseline characteristics between the two groups. Since follow-up of the study is less than 1 year, we will neither discount costs nor effects. To assess uncertainty surrounding the findings, 5000 bootstrapped replications will be calculated and presented on a cost-utility plane. In addition, ICUR acceptability curves will be presented and sensitivity analyses will be performed focusing on uncertainty surrounding most important cost items.

## Discussion

This study aims to investigate the effectiveness and cost-utility of a guided self-help exercise program to prevent or diminish speech, swallowing and neck and shoulder problems in patients treated with TL. Targeting these problems in patients treated with TL is of importance, since a major group of patients report such problems, which impact their quality of life and daily life [3–9].

Patients randomized to the guided self-help exercise program in our study will be asked to perform different flexibility exercises three times a day for 12 weeks. Based on a previous study in head and neck cancer patients undergoing (postoperative) (chemo)radiation [28], the guided self-help exercise program of ITwC is hypothesized to be feasible. In this study of Cnossen et al. [28], uptake of the exercise program “Head Matters” was high (i.e. 83 %) and adherence (i.e. patients exercising at least once a day during their treatment period) was adequate (i.e. 64 %).

The guided self-help exercise program evaluated in the present study is hypothesized to prevent or diminish speech, swallowing and neck and shoulder problems in patients treated with TL. Previous randomized controlled trials have shown beneficial findings of an exercise program in head and neck cancer patients [11, 15, 17–19, 23–25, 27]. One randomized controlled trial in head and neck cancer patients on the effectiveness of an exercise program consisting of five prophylactic swallowing exercises throughout (chemo)radiation showed beneficial effects on functional oral intake, eating in public and normalcy of diet compared to usual care [15]. Usual care involved referral to a speech therapist if swallowing problems were present after completion of (chemo)radiation. Besides, three randomized controlled trials on the effectiveness of an exercise program that included device-based (i.e. Therabite) exercises showed beneficial effects on residue after swallowing [24], maintenance of muscle composition [11], mouth opening [11, 23], salivary flow [11], tube dependency [11], trismus [23] and swallowing function [11, 23] in head and neck cancer patients compared to control care. In addition, previous trials in head and neck cancer patients treated with a neck dissection in general showed beneficial effects of progressive resistance exercises targeting shoulder functioning on shoulder rotation [17, 19, 27], shoulder pain and disability [17, 19], muscular endurance and strength [17].

However, a recent randomized controlled trial by Mortensen et al. [20] did not find any beneficial effect of a swallowing exercise program consisting of seven prophylactic swallowing exercises combined with standard individualized dietary advice among head and neck cancer patients. Potential explanations that were reported for the lack of beneficial effects may be the low to moderate adherence rate to the exercises and the limited statistical power due to high drop-out rates.

Although some studies thus have already been performed on the effectiveness of exercise programs in a heterogeneous group of head and neck cancer patients, the results are inconclusive. Our study is expected to add important information to the above mentioned literature. At first, our study will provide information on the beneficial effects in a more homogeneous group of head and neck cancer patients, namely patients treated with TL. As reported in the introduction section, patients treated with TL were often not included in previous studies and, consequently, previous findings may not be representable for patients treated with TL. In addition, our study combines exercises for speech, swallowing and neck and shoulder problems in one exercise program, thereby targeting the prevention or treatment of speech, swallowing as well as neck and shoulder problems in patients treated with TL, while previous programs only targeted swallowing or shoulder problems. Another strength of our study is that special attention will be paid to improving adherence to the guided self-help exercise program. Patients will be asked to fill in a diary on the number of times practiced each day and will be contacted on a weekly basis by the speech pathologist or nurse practitioner/specialist by e-mail or telephone in order to motivate the patient and ask the patient on his/her exercise performance. Furthermore, due to these weekly contact moments in combination with the provision of written instructions, images and videos of the prescribed exercises and the relatively easy to learn exercises which bring negligible risks, patients only have to visit the medical center once for instructions on the prescribed exercises. This will possibly reduce drop-out of patients of the guided self-help exercise program.

In addition to focusing on clinical effectiveness, this study will also evaluate the cost-utility of a guided self-help exercise program. One previous study by Retèl et al. [45] reported on the cost-utility of a preventive swallowing exercise program and found that the exercise program had a probability of 83 % to be cost-effective at a willingness to pay value of €20,000/QALY. Performing economic evaluations are of importance, since this provides knowledge on the value for money of health care interventions, which is important knowledge when deciding on the implementation of interventions in clinical practice. A potential limitation of this study may be that patients in the control group were provided with the self-care education program of ITwC, while no control group without intervention was included. This design was chosen, since we did want to give all TL patients the opportunity to benefit from the self-care education program. Since we aimed to assess the effectiveness and cost-utility of the guided self-help program on speech, swallowing and shoulder problems specifically, and the self-care education program is not expected to influence these outcomes, this is not expected to be harmful.

### Ethics and dissemination

This study will be conducted according to the principles of the Declaration of Helsinki (version, October 2013) and in accordance with the Medical Research Involving Human Subjects Act (WMO). Risks of participation in this study are negligible.

If this guided self-help exercise program is (cost)effective for patients treated with TL, the next step will be to focus on implementation of this program in clinical practice. The collaboration with the Dutch Head and Neck Oncology Cooperative Group (NWHHT) [46] for this study and the involvement of the patients association for patients treated with TL (NSvG) [47] in the development process is expected to facilitate implementation of the guided self-help exercise program in clinical care. The NWHHT, the Paramedical Dutch Head and Neck Oncology Cooperative Group (PWHHT) and the NSvG will be informed about the findings of this study in order to facilitate implementation of this intervention in all centers in which patients with TL are treated. In addition, guideline committees will be informed and advised to adapt (inter)national guidelines on laryngeal cancer. Also, results will be published in peer-reviewed scientific journals.

## Abbreviations

CVZ, Dutch Health Care Insurance Board; EORTC QLQ-C30, EORTC core quality of life questionnaire; EORTC QLQ-H&N35, EORTC head & neck cancer specific quality of life questionnaire; EORTC, European Organization for Research and Treatment of Cancer; EQ-5D, EuroQoL-5 dimensions; ICUR, incremental cost-utility ratio; iMCQ, iMTA medical consumption questionnaire; iPCQ, iMTA productivity cost questionnaire; ITwC, In Tune without Cords; NSvG, patients association for patients treated with total laryngectomy; NWHHT, Dutch Head and Neck Oncology Cooperative Group; PAM, patient activation measure; PWHHT, Paramedical Dutch Head and Neck Oncology Cooperative Group; QALY, quality-adjusted life year; SDQ, shoulder disability questionnaire; SHI, speech handicap index; SWAL-QOL, swallowing quality of life questionnaire; TL, total laryngectomy; WMO, Medical Research Involving Human Subjects Act.
